# First Outpatient Evaluation of a Tubeless Automated Insulin Delivery System with Customizable Glucose Targets in Children and Adults with Type 1 Diabetes

**DOI:** 10.1089/dia.2020.0546

**Published:** 2021-06-02

**Authors:** Gregory P. Forlenza, Bruce A. Buckingham, Sue A. Brown, Bruce W. Bode, Carol J. Levy, Amy B. Criego, R. Paul Wadwa, Erin C. Cobry, Robert J. Slover, Laurel H. Messer, Cari Berget, Susan McCoy, Laya Ekhlaspour, Ryan S. Kingman, Mary K. Voelmle, Jennifer Boyd, Grenye O'Malley, Aimee Grieme, Kaisa Kivilaid, Krista Kleve, Bonnie Dumais, Todd Vienneau, Lauren M. Huyett, Joon Bok Lee, Jason O'Connor, Eric Benjamin, Trang T. Ly

**Affiliations:** ^1^ Barbara Davis Center for Diabetes, University of Colorado School of Medicine, Aurora, Colorado, USA.; ^2^ Department of Pediatrics, Division of Pediatric Endocrinology, Stanford University, Stanford, California, USA.; ^3^ Division of Endocrinology and Medicine, University of Virginia, Charlottesville, Virginia, USA.; ^4^ Atlanta Diabetes Associates, Atlanta, Georgia, USA.; ^5^ Icahn School of Medicine at Mount Sinai, New York, New York, USA.; ^6^ Department of Pediatric Endocrinology, Park Nicollet Clinic, International Diabetes Center at Park Nicollet, Minneapolis, Minnesota, USA.; ^7^ Covance, Inc., Princeton, New Jersey, USA.; ^8^ Insulet Corporation, Acton, Massachusetts, USA.

**Keywords:** Automated insulin delivery, Omnipod, Tubeless insulin pump, Artificial pancreas, Closed-loop

## Abstract

***Background:*** The objective of this study was to assess the safety and effectiveness of the first commercial configuration of a tubeless automated insulin delivery system, Omnipod^®^ 5, in children (6–13.9 years) and adults (14–70 years) with type 1 diabetes (T1D) in an outpatient setting.

***Materials and Methods:*** This was a single-arm, multicenter, prospective clinical study. Data were collected over a 14-day standard therapy (ST) phase followed by a 14-day hybrid closed-loop (HCL) phase, where participants (*n* = 36) spent 72 h at each of three prespecified glucose targets (130, 140, and 150 mg/dL, 9 days total) then 5 days with free choice of glucose targets (110–150 mg/dL) using the Omnipod 5. Remote safety monitoring alerts were enabled during the HCL phase. Primary endpoints were difference in time in range (TIR) (70–180 mg/dL) between ST and HCL phases and proportion of participants reporting serious device-related adverse events.

***Results:*** Mean TIR was significantly higher among children in the free-choice period overall (64.9% ± 12.2%, *P* < 0.01) and when using a 110 mg/dL target (71.2% ± 10.2%, *P* < 0.01), a 130 mg/dL target (61.5% ± 7.7%, *P* < 0.01), and a 140 mg/dL target (64.8% ± 11.6%, *P* < 0.01), and among adults using a 130 mg/dL target (75.1% ± 11.6%, *P* < 0.05), compared to the ST phase (children: 51.0% ± 13.3% and adults: 65.6% ± 15.7%). There were no serious device-related adverse events reported during the HCL phase, nor were there episodes of severe hypoglycemia or diabetic ketoacidosis.

***Conclusion:*** The Omnipod 5 System was safe and effective when used at glucose targets from 110 to 150 mg/dL for 14 days at home in children and adults with T1D.

## Introduction

Advances in diabetes technology have radically transformed the treatment paradigm for type 1 diabetes (T1D), yet, glycemic outcomes continue to be suboptimal, and the burden of disease is high.^1^ Automated insulin delivery or hybrid closed-loop (HCL) systems have proven to be safe and effective for patients with T1D, demonstrating marked improvement in the percentage of time in target range, 70–180 mg/dL, by 10%–11% or 2.6 h more per day compared to sensor-augmented pump therapy^2,3^ and reduction in hypoglycemia at various thresholds. These systems utilize a continuous glucose monitor (CGM), insulin pump, and algorithm to automatically adjust insulin delivery.

Despite the availability of at least three commercial HCL systems^3–5^ in the United States and Europe, widespread adoption of diabetes technology is limited by cost,^6,7^ enduring user satisfaction,^8^ device interoperability, and manufacturing precision.^9^ Barriers to device adoption and long-term success are well recognized,^6,7,10^ and there is a critical need for alternative therapies that reduce the unpredictable nature of glycemic variability as well as the psychological burden of this lifelong disease.^11^

The Omnipod^®^ 5 Automated Insulin Delivery System (Insulet Corporation, Acton, MA) is the first wearable, on-body, tubeless automated insulin delivery system. Its unique design offers benefits aimed at improving user satisfaction and reducing burden. Design of the Omnipod 5 System has been enabled by improvements in sensor accuracy, microprocessor speed, memory storage, secure communication through Bluetooth^®^ wireless technology, software development, and the miniaturization of insulin pump technology.

This HCL system consists of a tubeless insulin pump (“Pod”) and the Dexcom G6^®^ (Dexcom, Inc., San Diego CA), an interoperable CGM system, which provides automated insulin delivery with customizable glucose targets from 110 to 150 mg/dL, adjustable by time of day to allow for therapy personalization. The Omnipod 5 algorithm, previously known as the Horizon algorithm, has been evaluated in several clinical studies.^12–15^

The objective of this study was to demonstrate the safety and effectiveness of the Omnipod 5 System in children and adults with T1D at each of three prespecified glucose targets (130, 140, and 150 mg/dL), followed by a period of free choice of glucose targets, between 110 and 150 mg/dL. This was the first outpatient study of the commercial configuration of the Omnipod 5 System.

## Materials and Methods

### Study design

This single-arm, multicenter, prospective clinical study consisted of a 14-day outpatient standard therapy (ST) phase, followed by a 14-day HCL phase. For the ST phase, participants that were not using the Dexcom G6 CGM for their usual diabetes care wore a blinded Dexcom G6 CGM while managing their diabetes at home per their usual routine (e.g., multiple daily injections, pump therapy, CGM). Participants already using the Dexcom G6 CGM at the time of study enrollment instead provided CGM glucose data from the most recent 14-day period that met the minimum criteria (i.e., 80% CGM use, ≥2016 CGM values).

Following the ST phase, participants were trained on the Omnipod 5 System and immediately transitioned to the HCL phase. Participants took part in target glucose challenges during the HCL phase, with ∼72 h spent at each of the higher glucose targets of 130, 140, and 150 mg/dL (“challenge days”). For the remaining 5 days of the HCL phase, participants were able to choose their desired target glucose level or combination of targets between 110 and 150 mg/dL (“free-choice period”) (Supplementary Fig. S1).

For the HCL phase, participants were divided into two groups. The initial group (*n* = 16) spent the first 2 days supervised in a hotel or rental house and then transitioned to an outpatient environment for the remaining 12 days. The other group (*n* = 20) carried out the HCL phase entirely in an outpatient environment after all participants in the first group had completed the hotel or rental house stay. The number and source (Pod, handheld device/app, CGM sensor) of reported device deficiencies were recorded. Following the HCL phase, participants were able to transition into the 3-month pivotal study.

### Study participants

Participants were recruited at six sites in the United States from outpatient clinics or local recruitment registries from December 2019 to January 2020. Key inclusion criteria for the study were age 6–70 years, T1D for ≥6 months, hemoglobin A1c (HbA1c) value <10%, and participant appropriateness for pump therapy and capability and willingness to adhere to study protocol. Key exclusion criteria were a history of severe hypoglycemia or diabetic ketoacidosis (DKA) in the past 6 months, current pregnancy or lactation, use of noninsulin antidiabetic medication other than metformin, or participation in another clinical study using an investigational drug or device in the 30 days before or during the present study (full inclusion and exclusion criteria are available in Supplementary Table S1). There were no minimum or maximum criteria for total daily insulin (TDI) dose or body weight, as the system did not have any operational limits for these parameters.

The study received Institutional Review Board approval. All participants signed an informed consent form approved by their respective Institutional Review Board. Parents or guardians signed the form on behalf of minors (<18 years) (Clinicaltrials.gov registration NCT04176731).

### Safety and monitoring

During the HCL phase, the study staff monitored participants remotely to allow for real-time assessment of safety. Investigators were alerted via text message and contacted the participant for follow-up when there were 60 min with no data, or when sensor glucose was <55, <70 mg/dL for 20 min, or >300 mg/dL for 1 h.

Adverse events that were assessed and followed until resolution through both phases of the study included hypoglycemic events (defined as severe hypoglycemia requiring the assistance of another person or resulting in a serious adverse event), hyperglycemic events (defined as requiring evaluation, treatment or guidance from intervention site, blood glucose ≥300 mg/dL and ketones >1 mmol/L, or hyperglycemia resulting in a serious adverse event), and DKA (defined as hyperglycemia with the presence of polyuria, polydipsia, nausea or vomiting, serum ketones >1.5 mmol/L or large/moderate urine ketones, arterial blood pH <7.30, venous pH <7.24, or serum bicarbonate <15, and treatment provided in a health care facility). Glucose levels outside of the normal range were not considered an adverse event unless the other criteria outlined above were also experienced.

Serious adverse events were defined as any medical occurrences that led to death, life-threatening illness or injury, permanent impairment of body function or structure, in-patient or prolonged hospitalization, or medical or surgical intervention to prevent those mentioned above. A Data and Safety Monitoring Board was established and conducted periodic safety reviews of study data, including unintended adverse device effects and adverse events irrespective of device relationship.

### Investigational device

**FIG. 1.**
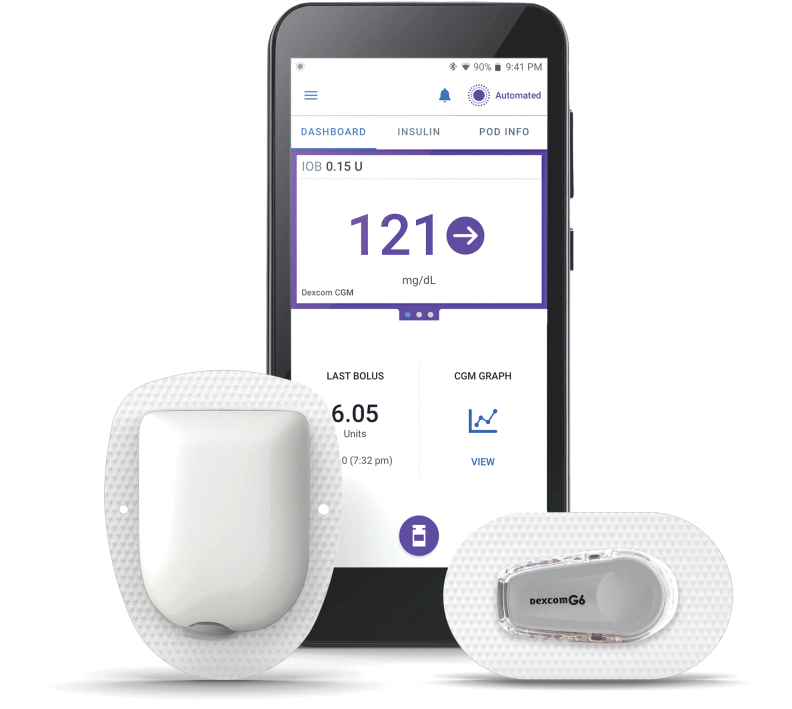
Components of the commercially intended Omnipod 5 Automated Insulin Delivery System. From left to right: (1) tubeless insulin pump (Pod) containing the automated insulin delivery algorithm (52 × 39 × 14.5 mm without adhesive, 26 g when empty); (2) Omnipod 5 application (app), pictured running on provided locked-down smartphone handheld device (144 × 67.6 × 12.4 mm, 165 g); (3) interoperable CGM (Dexcom G6, see Dexcom product documentation for additional information). The Pod is a lightweight, waterproof (IP28), self-adhesive insulin pump that delivers insulin through an automatically inserted cannula. The automated insulin delivery algorithm is built into the Pod, which receives glucose measurements every 5 min directly from the on-body CGM. The algorithm then commands the Pod to deliver microboluses every five minutes based on current and projected glucose values, with the goal of achieving and maintaining a set target glucose value. The user interacts with the system through the Omnipod 5 app, which communicates with the Pod through Bluetooth^®^ wireless technology. Actions performed using the app include: complete initial setup, activate and deactivate Pods, start Automated Mode, use the bolus calculator, deliver insulin boluses, enable the HypoProtect feature, view insulin delivery and CGM history, respond to system alerts and alarms, check Pod status, and adjust settable parameters. The app home screen (pictured) prominently displays the current CGM value and trend, as well as the amount of insulin on board, information about the last bolus, and a link to view the CGM history graph. The bolus calculator is accessed using the circular icon near the bottom of the screen. Since the algorithm runs on the Pod, and the Pod and CGM are both worn on-body and communicate directly, automated insulin delivery can continue uninterrupted even if the handheld device containing the app is not nearby. Copyrighted image used with permission. © 2020 Insulet Corporation. All rights reserved. CGM, continuous glucose monitor.

The Omnipod 5 Automated Insulin Delivery System (Omnipod 5) consists of the tubeless insulin pump (Pod) integrated with an interoperable CGM (Dexcom G6), and the Omnipod 5 mobile application (app) (Fig. 1). The Pod may be worn for up to 3 days and is filled with up to 200 U of U-100 rapid-acting insulin (minimum 85 U).

Each Pod contains a proprietary automated insulin delivery algorithm that delivers microboluses every five minutes based on current and projected glucose values to bring the glucose toward the target. To enable this process, the Pod receives glucose measurements directly from the CGM. Automated insulin delivery allows for real-time insulin attenuation and suspension with projected hypoglycemia and increased insulin delivery for projected hyperglycemia,^13^ in particular, to address postprandial hyperglycemia.

The user operates the system via the Omnipod 5 mobile app, which allows them to select basal profile, target glucose, and bolus settings, activate and deactivate the Pod, and connect the Pod with the glucose sensor. The mobile app runs on a compatible smartphone device. Participants in this study were required to use a provided locked-down smartphone device, which meant that all functions not needed for the Omnipod 5 System were disabled.

The system operates in two modes: Automated Mode for automated insulin delivery with CGM connected and Manual Mode, which delivers insulin at preprogrammed basal rates, both with and without CGM connected. Since the Pod and CGM are worn on-body and communicate directly, automated insulin delivery can continue uninterrupted even if the handheld device running the mobile app is not with the user or if its battery dies. The novel features of the Omnipod 5 System are intended to improve glycemic control and simplify diabetes management, ultimately to reduce the burden of care for the person with diabetes.

#### Algorithm description

The model predictive control (MPC) algorithm originated from Lee et al.^16^ It was adapted by Insulet Corporation for commercialization based on a series of clinical studies with physiological stressors, including missed meal boluses, meal over-boluses, high fat meals, and moderate-intensity exercise, in participants with T1D aged 2–68 years of age with wide-ranging insulin requirements (10–114 U/day).^17–19^

The Omnipod 5 algorithm drives insulin delivery toward a user-selected target glucose value, which can range from 110 to 150 mg/dL (6.1–8.3 mmol/L) in 10 mg/dL (0.55 mmol/L) increments. Target glucose values can be selected for different times of the day, e.g., a user may choose to have a lower target glucose at night of 110 mg/dL and a slightly higher target of 120 mg/dL during daytime hours, or they may prefer to have one target of 110 mg/dL for the entire day. For users with hypoglycemia unawareness, a higher target glucose may initially be desired if hypoglycemia is a concern^20^ and allows for a more gradual reduction of mean glucose. The system is an insulin-only HCL system and meal boluses are recommended to minimize postprandial hyperglycemia.

In addition to customized glucose control through user-selectable target glucose settings, users can activate the HypoProtect™ feature. Activation of HypoProtect temporarily sets the target glucose to 150 mg/dL, and additionally restricts insulin delivery. The design of this feature arose from studies of moderate-intensity exercise, during which users sought options to exercise safely and conveniently, whether or not they chose to take carbohydrates in advance.^14,17,18^ While originally conceived to address exercise, the combination of higher target glucose and restricted insulin delivery makes HypoProtect convenient for other periods of heightened insulin sensitivity or caution toward hypoglycemia (e.g., alcohol consumption, sleepover).

#### System initiation

Upon initiation of the system, the user, in consultation with their health care provider, creates a 24-h basal profile and bolus calculator settings, which include target glucose, insulin to carbohydrate ratio, correction factor, and maximum basal and bolus settings. These settings are similar to other commercially available insulin pumps, including Omnipod DASH^®^.^21^ Once connected to the Dexcom G6, via input of the transmitter serial number, the system can immediately be transitioned into Automated Mode, which enables automated insulin delivery.

Automated insulin delivery in the first Pod is initiated based on user-selected basal rate profiles. It then adapts over time by tracking insulin delivered by the system. As a safety measure, when the first Pod is activated, the algorithm is restrained until the second pod is activated. As each Pod is deactivated and a new one is activated, the system learns and adapts insulin delivery (also called “adaptive basal rate”) based on physiological needs and TDI delivered. Adaptation generally stabilizes after 2–3 Pod changes or 6–9 days. Automated insulin delivery is adjusted based on this adaptation and is decoupled from user-selected basal parameters. This alleviates the burden for the provider or user to continually adjust basal insulin profiles to achieve optimal closed-loop results. Additional system details can be found in Supplementary Table S2.

#### Bolus calculator

Another novel feature of the system is the bolus calculator, which incorporates both the CGM value and trend. CGMs provide information on both the current glucose value and the rate of change; however, bolus calculators in insulin pumps typically only allow the user to enter the glucose value and do not incorporate the trend.

While several sets of expert guidelines exist on how to adjust boluses based on CGM trend,^22–25^ the calculations must be made manually, are complex, and may be difficult for the user to remember and implement accurately. The Omnipod 5 bolus calculator allows the user to import the current CGM reading and trend, and then automatically increases or decreases the bolus amount based on the trend. The suggested bolus amount may be increased by up to 30%, or decreased by up to 100%, depending on the trend. This feature is available in both Automated and Manual Mode.

Omnipod 5 uses the user-set bolus calculator settings (insulin to carbohydrate ratio, correction factor, and target glucose) to calculate boluses in both Automated and Manual Mode. The duration of insulin action parameter, used to calculate insulin on board (IOB) from recent insulin delivered, is also customizable and impacts user-initiated bolus delivery (see Supplementary Table S2 for more details on IOB).

### Outcomes

The primary effectiveness outcome was the percentage of time spent with sensor glucose in target range (time in range [TIR]) (70–180 mg/dL)^26^ in the HCL phase during challenge days and free-choice days, compared to the ST phase. The secondary effectiveness endpoints were glucose metrics and insulin requirements during the HCL phase compared to the ST phase, including: mean, standard deviation (SD), and coefficient of variation of sensor glucose; percentage of time with sensor glucose in various ranges (i.e., <54, <70, >180, ≥250, ≥300 mg/dL); TDI; total daily basal insulin; and total daily bolus insulin.

The primary safety outcome of this study was the proportion of participants with serious device-related adverse events (e.g., life-threatening illness or injury, permanent impairment of body function or structure, unplanned or prolonged hospitalization, and death). The secondary safety outcomes were the proportion of participants with severe hypoglycemia or DKA during the HCL phase. Additional secondary outcomes included the percentage of time using automated insulin delivery as a proportion of overall study time and number and type of device deficiencies.

### Statistical analysis

This was a single-arm, multicenter, prospective study. The sample size was not hypothesis driven and was chosen to provide adequate information on the device's safety and performance. There were no hypotheses or success criteria associated with any of the primary or secondary endpoints for this study. The effectiveness endpoints were summarized for the modified intention to treat (mITT) analysis set. The mITT analysis set consists of enrolled participants who entered the HCL phase of the study successfully.

The data were stratified by study phase, where the data collected in the ST phase were compared to the data collected in the HCL phase. The percentage of time the participant spent in each specific glycemic range was calculated as the number of device readings in range, divided by the total number of device readings where the CGM value could be determined. For all summaries, device readings of “low” were assumed to be equal to 39 mg/dL and device readings of “high” were assumed to be equal to 401 mg/dL.

Standard statistical methods were used to analyze all data. All analyses were completed separately for children (aged 6–13.9 years) and for adults (aged 14–70 years). Continuous variables were summarized using descriptive statistics, including counts, mean, median, SD, minimum, and maximum. First and third quartiles were often presented. Categorical variables were summarized by frequencies and percentages. Unless explicitly stated otherwise, percentages utilized a denominator corresponding to the number of unique participants. No imputations for missing data were planned (or performed), and all analyses were based on available data only.

Paired *t*-tests were used to compare glycemic outcomes (e.g., mean, SD, and coefficient of variation of sensor glucose; percentage of time in specific glycemic ranges) and insulin use between the HCL and ST phases. Poisson regression using log link function, log of participant days as offset, and compound symmetry correlation structure was used to compare the average number of hypoglycemic and hyperglycemic events (as determined by glucose sensor) per person per day. All *P*-values were considered significant at a two-sided significance level of 0.05.

## Results

A total of 36 participants were enrolled in this study, 6 at each of the 6 investigational sites. The characteristics of the participants are reported in Table 1. All enrolled participants completed the study. Excluding the 72 h per person required at each of the higher targets 130–150 mg/dL, the percentage of total person-hours of HCL study time spent at the 110, 120, 130, 140, and 150 mg/dL targets was 56%, 10%, 23%, 3%, and 9% for children, respectively, and 49%, 33%, 6%, 5%, and 7% for adults.

**Table 1.** Characteristics of the Study Population

Characteristic	Children	Adults
	Aged 6–13.9 years (*n* = 18)	Aged 14–70 years (*n* = 18)
Age, years (range)	10.6 ± 1.8 (6.6–13.4)	35.0 ± 11.3 (20.5–65.5)
Diabetes duration, years (range)	5.2 ± 3.0 (0.9–10.3)	16.8 ± 11.6 (0.9–49.6)
Female, *n* (%)	12 (67)	13 (72)
White race, *n* (%)	18 (100)	15 (83)
Hispanic or Latino ethnic group, *n* (%)	1 (6)	3 (17)
Weight, kg (range)	40.3 ± 12.5 (24.1–76.2)	75.4 ± 12.9 (47.2–98.7)
HbA1c, % (range)	7.8 ± 0.9 (6.7–9.6)	7.1 ± 0.8 (5.9–8.9)
Standard therapy		
Insulin pump, *n* (%)	18 (100)	17 (94)
MDI, *n* (%)	0 (0)	1 (6)
History of insulin pump use		
Yes, *n* (%)	18 (100)	18 (100)
Duration, years (range)	2.9 ± 2.6 (0.4–8.4)	10.7 ± 6.8 (0.8–22.0)
History of CGM use, *n* (%)		
Yes, *n* (%)	18 (100)	18 (100)

Results are mean ± SD unless otherwise indicated.

CGM, continuous glucose monitoring; HbA1c, hemoglobin A1c; MDI, multiple daily injections; SD, standard deviation.

### Glycemic outcomes

The glycemic outcomes for the ST phase and the 5-day free-choice period of the HCL phase, overall and for the subset of participants who set their target glucose at 110 mg/dL, overall and overnight (00:00–6:00) for both age cohorts, are presented in Table 2.

**Table 2.** Glycemic Outcomes During the 5-Day Free-Choice Hybrid Closed-Loop Phase Overall and When Using a 110 mg/dL Glucose Target Compared to the 14-Day Standard Therapy Phase, Overall and Overnight and for Both Age Cohorts

						

Results are sensor glucose values, mean ± SD and/or median (IQR); SI conversion factor to convert glucose to mmol/L, multiply by 0.0555. ^*^P < 0.05, ^**^P < 0.01 determined using unadjusted two-sided paired *t*-tests comparing each HCL condition to ST. HCL, hybrid closed-loop; IQR, interquartile range; SI, international system of units; ST, standard therapy.

**FIG. 2.**
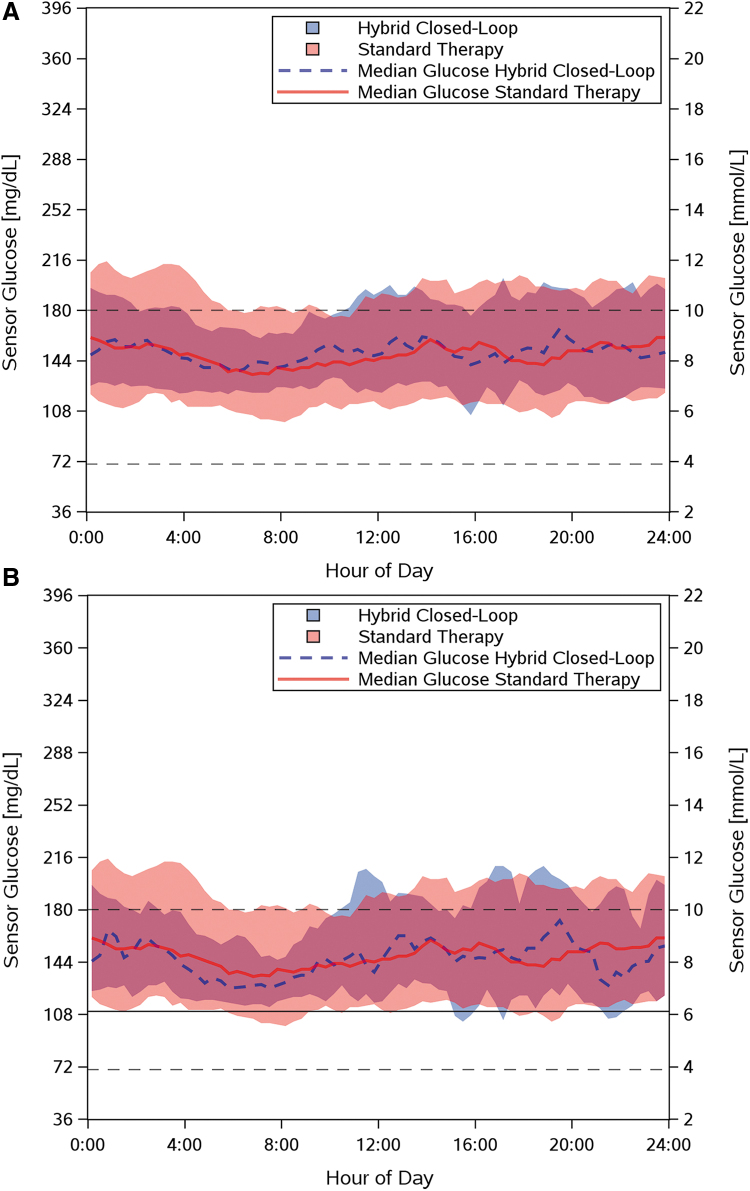

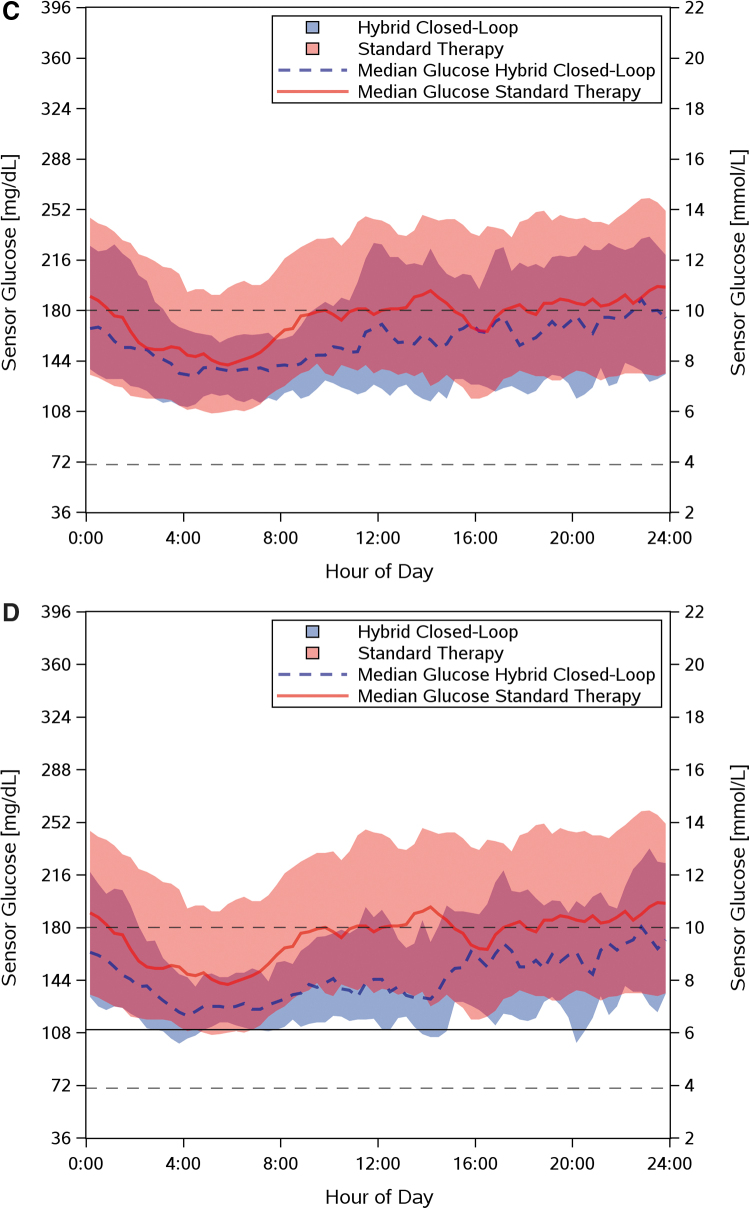
Interquartile plot of median sensor glucose profile over 24-h for **(A)** adults (age 14–70 years) during the HCL free-choice period overall (*n* = 18); **(B)** adults using the 110 mg/dL glucose target during the HCL free-choice period (*n* = 12); **(C)** children (age 6–13.9 years) during the HCL free-choice period overall (*n* = 18); and **(D)** children using the 110 mg/dL glucose target during the HCL free-choice period (*n* = 11), each compared to the median sensor glucose profile per age group from the 14-day ST phase. No prespecified glucose targets were used during the ST phase, as the ST was managed according to the patient's personal diabetes treatment goals. The data are presented as median (line) and interquartile range (shaded area) of sensor glucose per time of day across all participants and days. The target range of 70–180 mg/dL is indicated by black dashed lines, and the target glucose (if applicable) is indicated by a solid black line. HCL, hybrid closed-loop; ST, standard therapy.

In brief, for children, the mean TIR was significantly higher in the free-choice period overall and in the subset who set their target glucose to 110 mg/dL than during the ST phase both overall and overnight, but the corresponding differences were not significant among adults. Among children, the percentage of time spent with sensor glucose values <70, >180, ≥250, and ≥300 mg/dL decreased significantly from the ST phase to the free-choice period. Among adults, the percentage of time spent with sensor glucose values <54, <70, and ≥250 mg/dL decreased significantly from the ST phase to the free-choice period. Twenty-four-hour glucose profiles during the free-choice period overall and when a 110 mg/dL target was used compared to ST are illustrated in Figure 2. 

Separate data for participants with target glucose set at 120 mg/dL during the free-choice period of the HCL phase can be found in Supplementary Table S3. For adults, the percentage of time spent with sensor glucose values <70 mg/dL and glycemic variability were significantly lower when 120 mg/dL was set as the chosen target glucose than during the ST phase. Otherwise, there were no significant differences in sensor glucose values for the relatively small cohort that set 120 mg/dL as the target glucose.

Overall, the number of children with >60% and >70% TIR increased from 4 and 2 in the ST phase to 13 and 6 in the free-choice period of the HCL phase, respectively, out of 18 total. The number of adults with >70% TIR increased from 9 in the ST phase to 10 in the free-choice period of the HCL phase, out of 18 total.

The glycemic outcomes for the ST phase and the higher glucose targets used during the challenge days of the HCL phase, overall and overnight (00:00–6:00) for both age cohorts, are presented in Table 3.

**Table 3.** Glycemic Outcomes at the Specific Glucose Targets (130, 140, and 150 mg/dL) Used During 9 Challenge Days (at Least 3 Days at Each Target) of the Hybrid Closed-Loop Phase Compared to the 14-Day Standard Therapy Phase, Overall and Overnight and for Both Age Cohorts

								

Results are sensor glucose values, mean ± SD and/or median (IQR); SI conversion factor to convert glucose to mmol/L, multiply by 0.0555.

^a^ Data from additional usage of these higher targets during the free-choice period are also included for the outcomes reported in this table.

^b^ Due to the nonrandomized order of the higher targets, use of the 130 mg/dL target coincided with the initial Pod used by each participant. As described in “Investigational Device,” with the initial Pod the algorithm is constrained until the second Pod is activated. This may have resulted in a higher coefficient of variation with the initial Pod, which improved with subsequent Pods when the algorithm was fully activated.

^*^ *P* < 0.05, ^**^P < 0.01 determined using unadjusted two-sided paired *t*-tests comparing each HCL condition to ST.

Among children, the mean percentage of time spent outside of target range (<54, <70, >180, ≥250, ≥300) was significantly lower for most of the challenge-day targets than during the ST phase, and TIR was significantly higher when using the 130 and 140 mg/dL glucose targets. Among adults, the percentage of time spent in hypoglycemia (<54 and <70 mg/dL) was significantly lower at each of the glucose targets compared to during the ST phase, and TIR was significantly higher when using the 130 mg/dL target glucose. Twenty-four-hour glucose profiles for children and adults using the higher targets included in the challenge days of the HCL phase compared to ST are illustrated in Supplementary Figures S2 and S3.

Compared to the ST phase, participants experienced fewer episodes of hypoglycemia and hyperglycemia, defined according to sensor glucose values, per person per day during the HCL phase (Table 4), except for the number of hyperglycemic episodes per person per day among adults, which was not significantly different between the two phases.

**Table 4.** Average Number of Hypoglycemic and Hyperglycemic Events^a^ per Person per Day^b^ Detected by the Glucose Sensor During the ST and HCL Phases, by Age Cohort

Event type	Children				Adults			
	Aged 6–13.9 years (*n* = 18)				Aged 14–70 years (*n* = 18)			
	ST phase	HCL phase	Ratio HCL:ST	*P*^c^	ST phase	HCL phase	Ratio HCL:ST	*P*^c^
Hypoglycemia (<54 mg/dL)	0.137	0.056	0.41	0.0056	0.296	0.052	0.18	0.0005
Hypoglycemia (<70 mg/dL)	0.693	0.357	0.52	<0.0001	0.996	0.386	0.39	<0.0001
Hyperglycemia (>300 mg/dL)	1.076	0.686	0.64	0.0008	0.329	0.213	0.65	0.1589

^a^ An event was defined as three consecutive glucose sensor readings <54, <70, or >300 mg/dL. The end of an event was defined as three consecutive readings ≥54, ≥70, or ≤300 mg/dL, respectively. Additional provisions were made to handle any gaps in glucose sensor data appropriately.

^b^ Events per person per day was calculated as the number of events divided by the cumulative length of time in the appropriate phase (ST or HCL) across all participants.

^c^ *P*-value comparing ST to HCL determined based on Poisson regression using log link function, log of participant days as offset, and compound symmetry correlation structure.

### Insulin delivery outcomes

The average number of total units of insulin per day, per kg/day, and bolus insulin units per kg/day significantly decreased among children from the ST to the HCL phase (Table 5). Among adults, the average number of basal insulin units per day and per kg/day significantly decreased from the ST to the HCL phase. The average number of user-initiated boluses per day increased from 5.8 ± 2.3 to 8.0 ± 2.2 among adults (*P* = 0.0002).

**Table 5.** Insulin Use During Hybrid Closed-Loop and Standard Therapy Phases, by Age Cohort

Total daily dose of insulin	Children			Adults		
	Aged 6–13.9 years (*n* = 18)			Aged 14–70 years (*n* = 18)		
	ST	HCL	*P*^a^	ST	HCL	*P*^a^
Units/day						
Total	37.0 ± 15.6 (11.2, 67.7)	34.8 ± 16.3 (9.9, 66.8)	0.01	42.1 ± 10.9 (14.5, 56.5)	39.2 ± 12.5 (10.5, 74.4)	0.2
Basal	17.1 ± 11.0	16.7 ± 8.8	0.8	24.2 ± 8.3	18.2 ± 6.4	0.001
Bolus	19.8 ± 9.0	18.0 ± 8.3	0.1	19.0 ± 8.2	21.0 ± 7.1	0.2
Units/kg/day						
Total	0.90 ± 0.25 (0.46, 1.36)	0.83 ± 0.24 (0.41, 1.27)	0.03	0.56 ± 0.14 (0.19, 0.83)	0.53 ± 0.17 (0.14, 0.94)	0.2
Basal	0.41 ± 0.16	0.40 ± 0.11	0.8	0.32 ± 0.10	0.24 ± 0.08	0.002
Bolus	0.50 ± 0.19	0.44 ± 0.15	0.02	0.26 ± 0.12	0.29 ± 0.10	0.2
Boluses per day	8.4 ± 2.8	8.0 ± 2.2	0.5	5.8 ± 2.3	8.0 ± 2.2	0.0002

Results are mean ± SD (range).

^a^ *P*-value comparing ST to HCL determined using unadjusted two-sided paired *t*-tests.

### Safety outcomes

There were no serious device-related adverse events reported during the 14-day HCL phase, nor were there episodes of severe hypoglycemia or DKA as defined in the protocol (Table 6). There were four total reported adverse events during the HCL phase, including a skin infection that developed at a child's personal pump infusion site removed at the start of HCL, and among adults, one incidence each of prolonged hyperglycemia relating to a dislodged cannula, moderate ketosis, and unrelated shoulder pain. See Table 6 for additional details related to these events.

**Table 6.** All Adverse Events Reported During the 14-Day Hybrid Closed-Loop Phase, by Age Cohort

	Children		Adults	
	Aged 6–13.9 years (*n* = 18)		Aged 14–70 years (*n* = 18)	
	Events, *n*	Participants,* n *(%)	Events, *n*	Participants,* n *(%)
Total adverse events	1	1 (5.6)	3	3 (16.7)
Hypoglycemia^a^	0	0 (0.0)	0	0 (0.0)
Severe hypoglycemia^b^	0	0 (0.0)	0	0 (0.0)
DKA^c^	0	0 (0.0)	0	0 (0.0)
Prolonged hyperglycemia^d^	0	0 (0.0)	1	1 (5.6)
Hyperglycemia^e^	0	0 (0.0)	0	0 (0.0)
Other^f^	1	1 (5.6)	2	2 (11.1)

^a^ Hypoglycemia resulting in a serious adverse event but otherwise not meeting the definition of severe hypoglycemia.

^b^ Severe hypoglycemia requiring the assistance of another person due to altered consciousness, and requiring another person to actively administer carbohydrate, glucagon, or other resuscitative actions.

^c^ Hyperglycemia with the presence of polyuria, polydipsia, nausea or vomiting, serum ketones >1.5 mmol/L or large/moderate urine ketones, either arterial blood pH <7.30, venous pH <7.24, or serum bicarbonate <15, and treatment provided in a health care facility.

^d^ Meter blood glucose measuring ≥300 mg/dL and ketones >1.0 mmol/L; the single event reported lasted 2.7 h from onset to resolution and was due to a dislodged cannula.

^e^ Hyperglycemia requiring evaluation, treatment or guidance from intervention site, or hyperglycemia resulting in a serious adverse event but otherwise not meeting the definition of DKA or prolonged hyperglycemia.

^f^ The other adverse events reported in this study included a skin infection at the personal infusion site removed at start of HCL (children cohort), moderate ketosis, and unrelated shoulder pain.

DKA, diabetic ketoacidosis; HCL, hybrid closed-loop.

### System use

During the HCL phase, the percentage of time spent in automated mode was 97.3% ± 3.0% (82.1%–99.2%) (mean ± SD [range]), 97.3% ± 3.9% (82.1%–99.2%), and 97.2% ± 1.6% (93.2%–99.2%) for overall, children, and adults, respectively. The percentage of time spent in manual mode was 2.1% ± 2.2% (0.4%–12.4%), 2.2% ± 2.8% (0.5%–12.4%), and 2.1% ± 1.5% (0.4%–6.5%) for overall, children, and adults, respectively. Overall, there were 17 device deficiencies among 11 participants related to Pod, handheld device/app, and sensor function. Examples included Pod alarms resulting in deactivation, communication failure between Pod and app, app/handheld device errors requiring restart, and sensor errors requiring sensor change. None of the device deficiencies were associated with adverse events.

## Discussion

The results of this single-arm, multicenter, prospective clinical study demonstrated that use of the Omnipod 5 System with customizable glucose targets from 110 to 150 mg/dL was safe and effective over 14 days of outpatient use in adults and children with T1D. Automated insulin delivery was associated with greater time spent in the 70–180 mg/dL range and less time spent <70 mg/dL, compared to ST, under several conditions. Furthermore, participants experienced fewer hypoglycemic episodes compared to ST and no serious device-related adverse events, severe hypoglycemia, or DKA during Omnipod 5 use. The overnight mean glucose was lowest with the target glucose of 110 mg/dL (152 mg/dL in children and 149 mg/dL in adults) and highest with the target glucose of 150 mg/dL (181 mg/dL in children and 168 mg/dL in adults), suggesting that target level can affect the level of control, particularly in the overnight period.

The primary effectiveness outcome of this study was a greater percentage of TIR when using Omnipod 5 compared to the ST phase, with HCL results exceeding recent recommendations.^26^ An international consensus group recently recommended that TIR should be >70%, or >60% for those aged <25 years whose HbA1c target is 7.5%.^26^ The results demonstrate feasibility in achieving levels consistent with these guidelines. Encouragingly, increases in TIR have been shown to associate with lower rates of diabetes complications and lower HbA1c,^27–29^ with clinically significant benefits seen with each 5% increase in TIR.^27^ Results of this study concur with a meta-analysis of randomized controlled trials showing a 12.59% increase in TIR with the use of HCL systems compared with other continuous subcutaneous insulin infusion systems^30^ as well as other studies of adults and children who achieved greater TIR using HCL systems.^3–5,31–33^

Effective glucose control cannot come at a cost of greater hypoglycemia. Fear of hypoglycemia is a critical factor limiting optimal glycemic control^34,35^ since prolonged hypoglycemia during sleep can result in seizures, coma, and death.^36,37^ Ideally, <4% of time should be spent <70 mg/dL, and <1% of time should be spent with sensor glucose values <54 mg/dL.^26^ The percentage of time <70 and <54 mg/dL during both the free-choice and challenge days of the HCL phase were far below these recommendations. Further, the study results translate to a 2.5-fold and 5-fold reduction in number of hypoglycemic events per person per day (<54 mg/dL) for children and adults, respectively, in the HCL phase compared to the ST phase. These results compare favorably to previous research demonstrating reduced time spent in hypoglycemia when using HCL systems,^3–5,31,33^ but longer and larger studies are needed to confirm this comparison.

Efforts to minimize hyperglycemia are also important. In the present study, hyperglycemia among children was significantly reduced in the HCL phase compared to the ST phase when measured by events per person per day, and also by percentage of time during the free-choice days and with many of the challenge-day glucose targets. Such a finding has important clinical implications as hyperglycemia among children has been shown to impact brain development with worse glycemic control associated with reduced white matter and gray matter growth.^38^ It is evident from the present results that the Omnipod 5 System offers the potential to avoid extreme glucose values.

Glucose management should ideally be tailored to meet individual needs,^26^ making the ability to customize target glucose settings an essential feature of the Omnipod 5. For example, users may prefer to set a lower target glucose during times when tighter control is desired and a higher target glucose when hypoglycemia is a more pressing concern than hyperglycemia. For most of the higher challenge-day targets, the mean sensor glucose values were not significantly different from the ST phase; however, the use of these higher glucose targets during the study was essential to provide evidence on their safety.

Unexpectedly, in adults, the TIR was numerically highest with use of the 130 mg/dL target rather than in the subset who used the 110 mg/dL target. This result may be due to variations in lifestyle factors outside the control of the study that can have a significant impact on glycemic outcomes (ex. carbohydrate content of meals, number of omitted boluses, and physical activity levels), especially as the study took place over the winter holidays in the United States. In addition, the nonrandomized order of target glucose usage, short duration at each target glucose, and small sample size make it difficult to draw firm conclusions about the comparative efficacy at each target.

Overall, despite the use of the higher glucose targets during the challenge days, the percentage of time spent in hyperglycemia (>180 mg/dL) was not significantly different from the ST phase, and in fact, it was lower for several of the targets overall and overnight. This result is encouraging, as it shows that the algorithm successfully increased insulin delivery for projected hyperglycemia regardless of which target was used.

On the contrary, the free-choice period of the HCL phase was more representative of system use outside of study conditions as participants were able to choose which target glucose or combination of targets to use. Many participants chose the lowest target of 110 mg/dL and saw favorable results. In addition to reductions in overall time spent in hypoglycemia and hyperglycemia when using the 110 mg/dL target, mean sensor glucose values decreased in children from 185 ± 23 mg/dL, which is above the recommended target range of 70–180 mg/dL,^26^ to 155 ± 18 mg/dL. A mean glucose level of 155 mg/dL has been shown to correlate with an HbA1c of 7.0%,^39^ the HbA1c target for children recommended by ISPAD^40^ and ADA.^41^

This study took place under free-living conditions during the winter holidays, and therefore results may be reflective of real-world challenges to glycemic control. The participants fell into wide ranges of age and duration of diabetes and thus support the generalizability of the findings; however, the adult population already achieved very good outcomes in the ST phase, which may partly explain the lack of significant difference between ST and HCL for TIR and mean glucose in this age group. The notable improvement in the adult cohort was seen in the significant reduction in time in hypoglycemic ranges.

While this study provides insight into outcomes achieved with each target glucose, there is no one-size-fits-all recommendation for which setting to use. Instead, patients can work with their health care providers to choose a target glucose profile that best allows them to meet their individual goals. This tailored approach is an important benefit of customizable targets, especially since each situation and patient is unique.

In addition to the clinical benefits of the Omnipod 5 described here, the system has been designed to meet the user's desire for convenience and confident diabetes management.^42^ Although participants in this study were provided with a locked-down smartphone device with the Omnipod 5 mobile app, the commercially available system will also have the option to use a downloadable mobile app on the user's personal smartphone to control Pods. The ability to control devices from personal smartphones is a much-requested feature, which enables convenience and discreet insulin delivery.^42^

Either choice of handheld device provides automatic flow of data to the cloud-based data management system. This functionality allows caregivers to remotely monitor insulin delivery data via mobile app, similar to the Omnipod VIEW™ app.^21^ In addition, data will also be automatically shared with cloud-based data management systems to allow providers to review both insulin delivery and sensor glucose patterns. The ability for providers to view patient data without manual uploads provides additional efficiency and convenience to users, especially as virtual diabetes care becomes more commonplace.^43^

Positively, participants in the present study used the system in Automated Mode for an average of 97.3% of the total HCL phase duration, enabled by system design and usability. This high rate bodes well for long-term efficacy since more time in HCL should correlate with better glycemic outcomes. This result corresponds to a feasibility study using an earlier generation of the Omnipod HCL system at 99.1% of time during a 36-h HCL phase.^15^ Studies on other HCL systems have shown lower time spent in automated mode at 75.8% for adolescents and 88% for adults in one 3-month study,^33^ 81%^31^ in another 3-month study, and at 90% in a 6-month study.^3^ The staff monitoring and follow-up during the present study and a shorter study period may account for the higher rates of adherence, and a longer study using the Omnipod 5 System will make for a more appropriate comparison.

The short duration of the HCL phase and the small sample size are notable limitations of this study. Participants spent only 14 days using the Omnipod 5 System, of which 9 were challenge days with glucose targets set higher than might have been chosen otherwise. Also, as this was the first outpatient study of the system, 24-h remote monitoring was included, which may have influenced the results. At the end of this study, all participants were transitioned to the pivotal study, which is a 3-month outpatient study with more than 200 patients enrolled and no 24-h remote monitoring, from which long-term findings can be gleaned (Clinicaltrials.gov registration NCT04196140).

In addition, only one of the participants in this study was using multiple daily injections as their ST modality. However, a feasibility study testing an earlier generation of the Omnipod HCL system demonstrated that previous multiple daily injection users were able to transition successfully.^12^

Finally, this trial took place in late December, when many Americans celebrate holidays and the New Year. As a result, patients may have been partaking in dietary and physical activities not typical of their usual lifestyles, which demonstrate the use of the Omnipod 5 System in more exceptional circumstances.

## Conclusion

This study was the first to test the commercial configuration of the Omnipod 5 Automated Insulin Delivery System in a cohort of free-living participants. The results provide evidence that the Omnipod 5 System is safe during the day and overnight and performed well for use in patients with T1D aged 6–70 years at all target glucose levels.

## Supplementary Material

Supplemental data
