# Supplementary material for: First Outpatient Evaluation of a Tubeless Automated Insulin Delivery System with Customizable Glucose Targets in Children and Adults with Type 1 Diabetes
Source: Diabetes Technol Ther. 2021 Jun 2;23(6):410–24. doi: 10.1089/dia.2020.0546 (PMC8215410; doi:10.1089/dia.2020.0546)
Supplement: Supplemental data [file Supp_Data.pdf]

## Supplemental Material

This appendix has been provided by the authors to give readers additional information about their work.

Supplement to: Forlenza, Buckingham, Brown, et al. First outpatient evaluation of a tubeless automated insulin delivery system with customizable glucose targets in children and adults with type 1 diabetes. *Diabetes Technology and Therapeutics.* 2021.

# Contents


Supplemental Material ........ 1

Acknowledgements ........ 2

Supplemental Tables ........ 3

**Supplemental Table S1**: Inclusion and Exclusion Criteria ........ 3

**Supplemental Table S2:** Additional information on the Omnipod 5 System ........ 5

**Supplemental Table S3:** Glycemic outcomes during the 5-day free-choice HCL phase for participants with glucose targets set at 120mg/dL compared to the 14-day ST phase, overall and overnight and for both age cohorts ........ 9

Supplemental Figures ........ 11

**Supplemental Figure S1**. 14-day hybrid closed-loop (HCL) phase timeline illustrating the sequence of glucose targets used, followed by the free-choice period. Following the free choice period, participants continued into the 3-month pivotal study ........ 11

**Supplemental Figure S2**: Interquartile plot of median sensor glucose profile over 24-hours for adults (age 14 to 70 years, n=18) during the HCL target challenge-days period using the glucose targets: (A) 130mg/dL; (B) 140mg/dL; and (C) 150mg/dL compared to ST.. ........ 12

**Supplemental Figure S3**: Interquartile plot of median sensor glucose profile over 24 hours for children (age 6 to 13.9 years, n=18) during the HCL target challenge-days period using the glucose targets: (A) 130mg/dL; (B) 140mg/dL; and (C) 150mg/dL compared to ST. ........ 13

## Acknowledgements

The authors would like to acknowledge the contributions of the below-named team members, without whose efforts the study would not have been possible.

**Barbara Davis Center for Diabetes, University of Colorado School of Medicine, Aurora, CO**
Emily Jost, Lindsey Towers, Samantha Lange, Estelle Escobar, Angela Karami

**Department of Pediatrics, Division of Pediatric Endocrinology, Stanford University, Stanford, CA**
Lisa Norlander, Liana Hsu

**Division of Endocrinology and Medicine, University of Virginia, Charlottesville, VA**
Emma Emory, Emma Seago, Mary Oliveri

**Atlanta Diabetes Associates, Atlanta, GA**
Brooke Narron

**Icahn School of Medicine at Mount Sinai, New York, NY**
Camilla Levister, David Lam, Selassie Ogyaadu, Dushyanthy Arasaratnam, Emily Nosova

**Insulet Corporation, Acton, MA**
Nikia Trinward, Rachel McElligott, Tanya Meletlides, Maria Urbina, Yibin Zheng, Steve Cardinali, Connor Gullifer, Jon Hardy, Alex Nguyen, Brenda Ferris, Leslie Barrett

## Supplemental Tables

**Supplemental Table S1**: Inclusion and Exclusion Criteria

| |
|---|
| ***Inclusion Criteria***<br>Subjects must meet all of the following criteria to be included in the study:<br>1. Age at time of consent/assent 6-70 years<br>2. Subjects aged < 18 years must be living with parent/legal guardian<br>3. Diagnosed with type 1 diabetes for at least 6 months. Diagnosis is based on investigator's clinical judgment.<br>4. Deemed appropriate for pump therapy per investigator's assessment taking into account previous history of severe hypoglycemic and hyperglycemic events, and other comorbidities<br>5. Investigator has confidence that the subject can successfully operate all study devices and is capable of adhering to the protocol<br>6. Willing to use only the following types of insulin during the study: Humalog, Novolog, Admelog, or Apidra during the study<br>7. Must be willing to set target glucose between 130-150 mg/dL each for approximately 72-hours on predefined days during the hybrid closed-loop phase<br>8. Must be willing to extend their participation into the pivotal study if they continue to meet the protocol criteria<br>9. Willing to wear the system continuously throughout the study<br>10. A1C <10% at screening visit<br>11. Must be willing to use the Dexcom App on the Omnipod Horizon™ PDM as the sole source of Dexcom data (with the exception of the Dexcom Follow App) during the hybrid closed-loop phase<br>12. Subjects scoring ≥ 4 on the Clarke Questionnaire must agree to have an overnight companion, defined as someone who resides in the same home or building as the study subject and who can be available overnight<br>13. Able to read and speak English fluently<br>14. Subject must be in an AT&T covered area<br>15. Willing and able to sign the Informed Consent Form (ICF) and/or has a parent/guardian willing and able to sign the ICF. Assent will be obtained from pediatric and adolescent subjects aged < 18 years per State requirements. |
| ***Exclusion Criteria***<br>Subjects who meet any of the following criteria will be excluded from the study:<br>1. A medical condition, which in the opinion of the investigator, would put the subject at an unacceptable safety risk<br>2. History of severe hypoglycemia (as defined in Section 11.3.3) in the past 6 months<br>3. History of DKA (as defined in Section 11.3.4) in the past 6 months, unrelated to an intercurrent illness, infusion set failure or initial diagnosis<br>4. Diagnosed with sickle cell disease<br>5. Diagnosed with hemophilia or any other bleeding disorders<br>6. Plans to receive blood transfusion over the course of the study<br>7. Currently diagnosed with anorexia nervosa or bulimia<br>8. Acute or chronic kidney disease (e.g. estimated GFR < 45) or currently on hemodialysis<br>9. History of adrenal insufficiency<br>10. Has taken oral or injectable steroids within the past 8 weeks or plans to take oral or injectable steroids during the course of the study<br>11. Unable to tolerate adhesive tape or has any unresolved skin condition in the area of sensor or pump placement<br>12. Plans to use insulin other than U-100 insulin intended for use in the study device during the course of the study<br>13. Use of non-insulin anti-diabetic medication other than metformin (e.g. GLP1 agonist, SGLT2 inhibitor, DPP-4 inhibitor, pramlintide)<br>14. Current or known history of coronary artery disease that is not stable with medical management, including unstable angina, or angina that prevents moderate exercise despite medical management, or a history of |

myocardial infarction, percutaneous coronary intervention, or coronary artery bypass grafting within the previous 12 months.

15. For subjects >50 years old or with diabetes duration >20 years, abnormal electrocardiogram consistent with increased risk of arrhythmia, ischemia, or prolonged $QT_c$ interval (> 450 ms)

16. Thyroid Stimulating Hormone (TSH) is outside of normal range with clinical signs of hypothyroidism or hyperthyroidism

17. Pregnant or lactating, or is a woman of childbearing potential and not on acceptable form of birth control (acceptable includes abstinence, condoms, oral/injectable contraceptives, IUD or implant)

18. Participation in another clinical study using an investigational drug or device within the preceding 30-days or intends to participate during the study period

19. Unable to follow clinical protocol for the duration of the study or is otherwise deemed unacceptable to participate in the study per the investigator's clinical judgment.

**Supplemental Table S2:** Additional information on the Omnipod 5 System

Table S2.1: User-settable parameters affecting insulin delivery and their functions in Manual and Automated modes

<table>
<tr><th>Parameter</th><th>Available Settings</th><th>Manual Mode</th><th>Automated Mode</th></tr>
<tr><td>Basal Program</td><td>Up to 24 segments per basal program, from 0 U/h to user-set maximum basal rate in 0.05 U/h increments</td><td>Basal insulin is delivered according to the active basal program</td><td>The basal program is used only once at system initiation, to determine the user's adaptive basal rate for automated insulin delivery<br>1. The adaptive basal rate is initially determined from the basal program that is used to activate the first Pod<br>2. The adaptive basal rate is updated with each subsequent Pod based on total insulin delivery history<br><u>NOTE</u>: The user-set basal program is only used during system initiation. Further changes to user-set basal program after system initiation will have no effect on insulin delivery during Automated Mode</td></tr>
<tr><td>Target Glucose</td><td>Maximum of 8 segments per day from 110 to 150 mg/dL in 10 mg/dL increments</td><td>Bolus Calculator uses difference between current glucose and target glucose when calculating a user-initiated correction bolus</td><td>The algorithm continuously adjusts insulin delivery above or below the adaptive basal rate to bring the sensor glucose toward the target glucose. During Automated Mode, it is the primary parameter that affects automated insulin delivery.<br><br>Bolus Calculator uses difference between current glucose and target glucose when calculating a user-initiated correction bolus</td></tr>
<tr><td>HypoProtect</td><td>Can be turned on for 1-72 h at a time</td><td>Not available</td><td>While active, the algorithm delivers insulin to a target glucose of 150 mg/dL (instead of the user's target glucose setting), reduces insulin delivery, and restricts maximum insulin delivery</td></tr>
<tr><td>Duration of Insulin Action</td><td>2 to 6 h in 30-minute increments</td><td colspan="2">Used to calculate insulin on board (IOB) remaining from past meal and correction boluses (not used for Omnipod 5 Software IOB from basal delivery)</td></tr>
<tr><td>Correction Factor</td><td>Up to 8 segments per day, 1 to 400 mg/dL in 1 mg/dL increments</td><td colspan="2">Used by Bolus Calculator to calculate suggested amount for user-initiated correction boluses. For previous pump users, this setting may need to be adjusted to optimize outcomes in Automated Mode.</td></tr>
<tr><td>Insulin to Carbohydrate Ratio</td><td>Up to 8 segments per day, 1 to 150 g CHO/U in 0.1 g CHO/U increments</td><td colspan="2">Used by Bolus Calculator to calculate suggested amount for user-initiated meal boluses. For previous pump users, this setting may need to be adjusted with Automated Mode use since if users come in at or below their target glucose prior to a meal, the amount of active insulin in their body will likely be less compared to their previous therapy.</td></tr>
</table>

| Parameter | Available Settings | Manual Mode | Automated Mode |
| --- | --- | --- | --- |
| **Correct Above Value** | Up to 8 segments per day from target BG to 200 mg/dL in 1 mg/dL increments | When the user initiates a bolus with the Bolus Calculator, a correction bolus will only be suggested if the current glucose value is above the Correct Above Value | |
| **Minimum BG for Calcs** | 50 to 70 mg/dL in 1 mg/dL increments | The Bolus Calculator will be disabled when current glucose value is below the Minimum BG for Calcs | |
| **Reverse Correction** | On or off | Determines whether reverse corrections will be suggested when the user initiates a bolus with the Bolus Calculator. Reverse corrections reduce the meal bolus amount when the current glucose value is below the target glucose. | |
| **Maximum Bolus Size** | 0.05 to 30 U | Defines the upper limit for a bolus | |
| **Extended Bolus** | On or off | Determines if extended boluses can be delivered in Bolus Calculator | *Not applicable* - Extended boluses are not available in automated mode |
| **Maximum Basal Rate** | 0.05 – 30 U/h | Sets the upper limit of any basal insulin rate that can be used in Manual Mode | *Not applicable* - The maximum allowable amount that can be given at any time while in Automated Mode varies from person to person and is based on their insulin needs and past deliveries. The maximum amount that can be delivered in Automated Mode is independent of the maximum basal rate setting. |

Table S2.2: Insulin on Board in the Omnipod 5 System. All types of IOB listed are tracked in both Automated and Manual modes.

| Parameter | Definition | How it is calculated | How it is used in Bolus Calculator |
|---|---|---|---|
| **Meal IOB** | Insulin remaining in the body from previous meal boluses | Decays according to duration of insulin action setting | Meal IOB is subtracted from the suggested correction bolus. Meal IOB is never subtracted from a meal bolus |
| **Correction IOB** | Insulin remaining in the body from previous correction boluses | Decays according to duration of insulin action setting | Correction IOB is subtracted from the suggested correction bolus AFTER any meal IOB has been subtracted. Any remaining Correction IOB is subtracted from the suggested meal bolus‡ |
| **Omnipod 5 Software IOB†** | Insulin remaining in the body from basal or automated insulin delivery above the user's adaptive basal rate | Decays according to a variable curve and is not dependent on the duration of insulin action setting | For the purpose of bolus calculations, Omnipod 5 Software IOB is added to the Correction IOB |
| **Omnipod 5 System IOB** | IOB displayed on the system home screen | Sum of the correction IOB, meal IOB, and Omnipod 5 Software IOB i.e. all insulin delivered by the system | Each component affects boluses differently according to the above three rows |

‡The Bolus Calculator will only adjust a meal bolus for IOB if a glucose value has been entered

†Note: the Omnipod 5 Software IOB represents IOB from basal or automated insulin delivery and accumulates in both Manual Mode and Automated Mode. The concept of IOB from basal insulin delivery is novel and results from the system's ability to learn the user's insulin needs over time. Through this functionality, the system can determine when a basal insulin delivery rate is greater than the user's anticipated basal need. Users may require training on this concept, as they may not expect to see IOB accumulate from basal delivery in Manual Mode.

Table S2.3: Automated insulin delivery with the Omnipod 5 System

| When sensor glucose is… | System Action |
|---|---|
| **Above Target Glucose** | • Automated insulin delivery will generally increase when sensor glucose is predicted to be above the specified target glucose.<br>• If sensor glucose is trending downwards, automated insulin delivery may still decrease/pause even if sensor glucose value is above the target. |
| **Below Target Glucose** | • Automated insulin delivery will generally decrease or pause when sensor glucose is predicted to be below the specified target glucose.<br>• If sensor glucose is trending upwards, automated insulin delivery may still increase even if sensor glucose value is below the target. |
| **<60 mg/dL** | The system will always pause insulin delivery when sensor glucose is <60 mg/dL |

**Supplemental Table S3:** Glycemic outcomes during the 5-day free-choice HCL phase for participants with glucose targets set at 120mg/dL compared to the 14-day ST phase, overall and overnight and for both age cohorts

| **Age Group** | **Children Aged 6-13.9 years (n=18)** | | **Adults Aged 14-70 years (n=18)** | |
|---|---|---|---|---|
| **Study Phase** | **ST** | **HCL** | **ST** | **HCL** |
| **Glucose Target (mg/dL)** | **-** | **120** | **-** | **120** |
| **N** | **18** | **3** | **18** | **7** |
| **Overall** | | | | |
| Mean sensor glucose, mg/dL | 185 ± 23 | 170 ± 16 | 157 ± 26 | 160 ± 11 |
| Standard deviation, mg/dL | 71 ± 15 | 72 ± 7 | 56 ± 11 | 46 ± 13** |
| Coefficient of variation, % | 38 ± 6 | 43 ± 3 | 36 ± 5 | 29 ± 7** |
| Percentage time in glucose range, % | | | | |
| <54 mg/dL | 0.4 ± 0.7<br>0.1 (0.0, 0.5) | 0.2 ± 0.1<br>0.2 (0.0, 0.3) | 0.8 ± 1.0<br>0.6 (0.1, 1.1) | 0.0 ± 0.0<br>0.0 (0.0, 0.0) |
| <70 mg/dL | 2.3 ± 3.0<br>1.1 (0.4, 2.8) | 0.9 ± 1.2<br>0.3 (0.2, 2.2) | 3.4 ± 3.0<br>2.6 (0.9, 5.1) | 0.4 ± 0.3*<br>0.4 (0.0, 0.6) |
| 70-180 mg/dL | 51.0 ± 13.3<br>48.5 (39.7, 57.3) | 66.8 ± 12.9<br>67.7 (53.5, 79.3) | 65.6 ± 15.7<br>70.1 (48.7, 76.4) | 70.9 ± 11.3<br>75.3 (64.8 80.6) |
| >180 mg/dL | 46.7 ± 13.8<br>48.4 (41.2, 59.2) | 32.3 ± 11.9<br>32.1 (20.5, 44.3) | 30.9 ± 16.9<br>28.7 (19.2, 45.0) | 28.7 ± 11.2<br>24.7 (18.8, 35.2) |
| ≥250 mg/dL | 19.3 ± 8.8<br>21.4 (12.1, 26.6) | 14.4 ± 6.2<br>11.8 (10.0, 21.5) | 9.0 ± 8.4<br>6.8 (2.7, 16.4) | 5.2 ± 5.5<br>3.7 (1.7, 9.2) |
| ≥300 mg/dL | 8.3 ± 5.4<br>7.9 (4.1, 12.8) | 7.1 ± 2.1<br>8.1 (4.7, 8.5) | 2.5 ± 3.2<br>0.6 (0.1, 4.8) | 1.5 ± 2.9<br>0.0 (0.0, 3.3) |
| **Overnight (00:00-06:00)** | | | | |
| Mean sensor glucose, mg/dL | 170 ± 29 | 159 ± 11 | 161 ± 29 | 161 ± 27 |
| Standard deviation, mg/dL | 62 ± 21 | 60 ± 29 | 59 ± 15 | 43 ± 24** |
| Coefficient of variation, % | 37 ± 11 | 37 ± 16 | 37 ± 7 | 25 ± 10** |
| Percentage time in glucose range, % | | | | |
| <54 mg/dL | 1.0 ± 1.6<br>0.1 (0.0, 1.1) | 0.0 ± 0.0<br>0.0 (0.0, 0.0) | 1.3 ± 1.9<br>0.6 (0.0, 1.7) | 0.0 ± 0.0<br>0.0 (0.0, 0.0) |
| <70 mg/dL | 4.5 ± 6.7<br>1.2 (0.0, 8.2) | 0.0 ± 0.0<br>0.0 (0.0, 0.0) | 3.9 ± 3.3<br>3.1 (1.1, 6.5) | 0.1 ± 0.4<br>0.0 (0.0, 0.0) |
| 70-180 mg/dL | 55.6 ± 15.7<br>52.4 (43.2, 61.0) | 80.0 ± 10.2<br>82.7 (68.8, 88.5) | 62.9 ± 16.8<br>62.7 (52.4, 77.6) | 73.9 ± 16.8<br>67.6 (61.8, 92.4) |

| | | | | |
|---|---|---|---|---|
| >180 mg/dL | 39.9 ± 17.4<br>42.6 (28.5, 49.0) | 20.0 ± 10.2<br>17.3 (11.5, 31.3) | 33.2 ± 17.8<br>31.8 (17.7, 42.4) | 25.9 ± 17.0<br>32.4 (7.6, 38.2) |
| ≥250 mg/dL | 14.4 ± 10.4<br>11.6 (9.1, 24.9) | 9.5 ± 8.3<br>14.0 (0.0, 14.6) | 10.9 ± 10.6<br>5.8 (4.1, 17.3) | 7.8 ± 12.4<br>0.7 (0.0, 13.1) |
| ≥300 mg/dL | 5.5 ± 5.8<br>4.1 (0.5, 8.6) | 6.4 ± 6.1<br>6.9 (0.0, 12.1) | 3.6 ± 5.7<br>0.2 (0.0, 4.3) | 3.9 ± 8.6<br>0.0 (0.0, 4.4) |

Results are sensor glucose values, mean ± SD and/or median (IQR); SI conversion factor to convert glucose to mmol/L, multiply by 0.0555.
*$p<0.05$ **$p<0.01$ determined using unadjusted two-sided paired t-tests comparing HCL to ST.
**Abbreviations:** HCL, hybrid closed-loop; IQR, interquartile range, SD, standard deviation; SI, international system of units; ST, standard therapy.

## Supplemental Figures

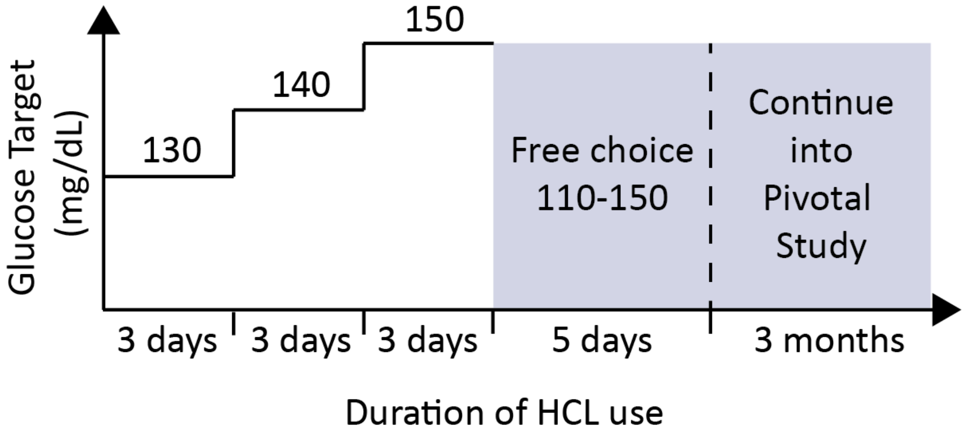


**Supplemental Figure S1**. 14-day hybrid closed-loop (HCL) phase timeline illustrating the sequence of glucose targets used, followed by the free-choice period. Following the free choice period, participants continued into the 3-month pivotal study.

**A.** Target Glucose 130mg/dL **B.** Target Glucose 140mg/dL **C.** Target Glucose 150mg/dL

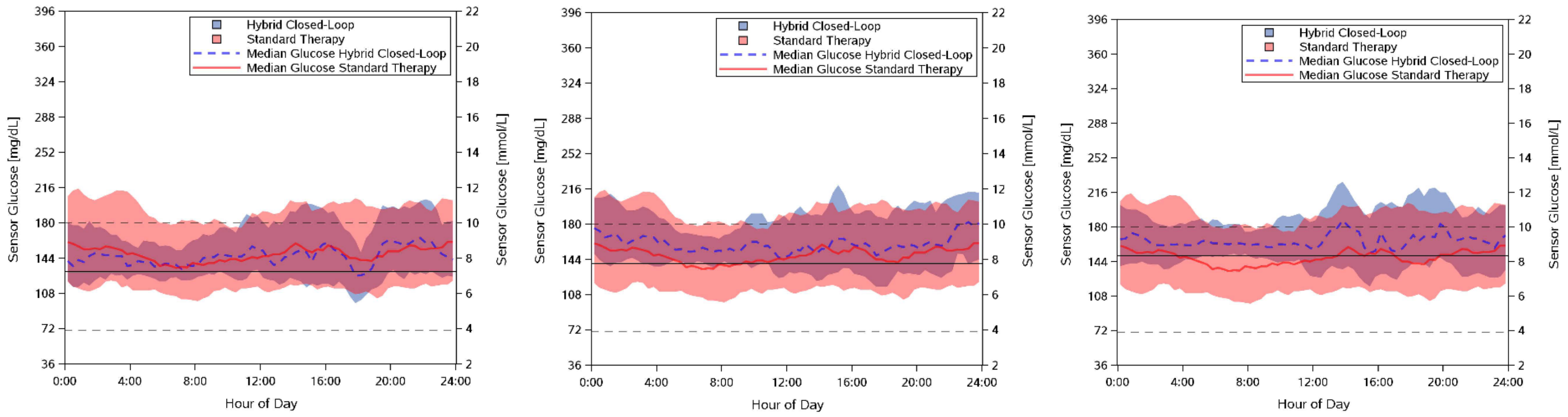


**Supplemental Figure S2**: Interquartile plot of median sensor glucose profile over 24-hours for adults (age 14 to 70 years, n=18) during the HCL target challenge-days period using the glucose targets: (A) 130mg/dL; (B) 140mg/dL; and (C) 150mg/dL compared to ST. The data are presented as median (line) and interquartile range (shaded area) of sensor glucose per time of day across all participants and days. The target range of 70-180 mg/dL is indicated by black dashed lines, and the target glucose is indicated by a solid black line. All three HCL targets were compared to the same 14-day ST period. No pre-specified glucose targets were used during the ST phase. The ST was managed according to the patient's personal diabetes treatment goals. Abbreviations: HCL, hybrid closed-loop; ST, standard therapy.

**A.** Target Glucose 130mg/dL **B.** Target Glucose 140mg/dL **C.** Target Glucose 150mg/dL

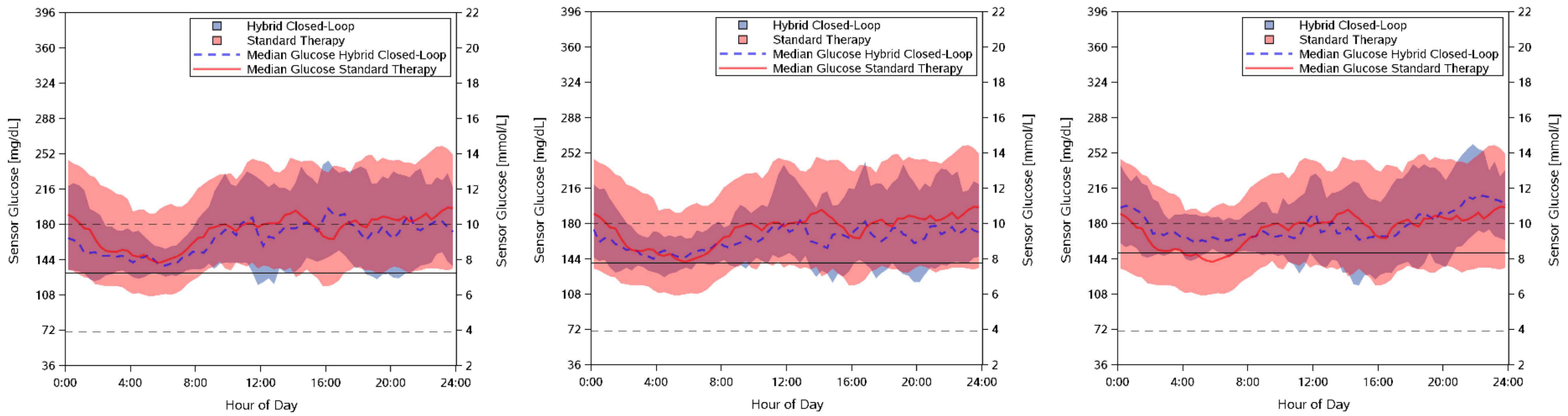


**Supplemental Figure S3**: Interquartile plot of median sensor glucose profile over 24 hours for children (age 6 to 13.9 years, n=18) during the HCL target challenge-days period using the glucose targets: (A) 130mg/dL; (B) 140mg/dL; and (C) 150mg/dL compared to ST. The data are presented as median (line) and interquartile range (shaded area) of sensor glucose per time of day across all participants and days. The target range of 70-180 mg/dL is indicated by black dashed lines, and the target glucose is indicated by a solid black line. All three HCL targets were compared to the same 14-day ST period. No pre-specified glucose targets were used during the ST phase. The ST was managed according to the patient's personal diabetes treatment goals. Abbreviations: HCL, hybrid closed-loop; ST, standard therapy.
